# Use of *Aeromonas* spp. as General Indicators of Antimicrobial Susceptibility among Bacteria in Aquatic Environments in Thailand

**DOI:** 10.3389/fmicb.2016.00710

**Published:** 2016-05-12

**Authors:** Masaru Usui, Chie Tagaki, Akira Fukuda, Torahiko Okubo, Chanchai Boonla, Satoru Suzuki, Kanako Seki, Hideshige Takada, Yutaka Tamura

**Affiliations:** ^1^School of Veterinary Medicine, Rakuno Gakuen UniversityEbetsu, Japan; ^2^Departments of Biochemistry, Chulalongkorn UniversityBangkok, Thailand; ^3^Center for Marine Environmental Studies, Ehime UniversityMatsuyama, Japan; ^4^Laboratory of Organic Geochemistry, Tokyo University of Agriculture and TechnologyFuchu, Japan

**Keywords:** *Aeromonas* spp., antimicrobial resistance, aquatic environments, tetracycline resistance, indicator bacteria

## Abstract

Antimicrobials are widely used, not only for treating human infections, but also for treatment of livestock and in fish farms. Human habitats in Southeastern Asian countries are located in close proximity to aquatic environments. As such, the human populations within these regions are at risk of exposure to antimicrobial resistant bacteria, and thereby disseminating antimicrobial resistance genes (ARGs). In this study, we collected water samples from 15 sites (5 sites in Chao Phraya River, 2 sites at the mouth of Chao Phraya River, 3 sites in Ta Chin River, and 5 sites at city canals) and 12 sites (6 sites at city canals; 2 sites at chicken farms; 2 sites at pig farms; and 2 samples from sites at pig farms, which were subsequently treated at a biogas plant) in Thailand in 2013 and 2014, respectively. In total, 117 *Aeromonas* spp. were isolated from the water samples, and these organisms exhibited various antimicrobial susceptibility profiles. Notably, there was a significant correlation between the environmental concentration of tetracyclines and the rates of tetracycline resistance in the isolated *Aeromonas* spp.; however, both the concentration and rates of tetracycline resistance in samples derived from pig farms were higher than those of samples harvested from other aquatic environments. These findings suggest that the high concentrations of antimicrobials observed in these aquatic environments likely select for ARGs. Furthermore, they indicate that *Aeromonas* spp. comprise an effective marker for monitoring antimicrobial resistance in aquatic environments.

## Introduction

The use of antimicrobials for treating/preventing bacterial infections is essential for promoting the health and welfare of humans and animals. As such, the emergence and spread of antimicrobial resistance is of global concern. To address these issues, the World Health Organization (WHO) and other international organizations require the development of programs for monitoring antimicrobial resistance in zoonotic bacteria, animal pathogens, and indicator bacteria derived from food-producing animals (Dehaumont, 2004; FAO/OIE/WHO, 2004).

Antimicrobials are widely used, not only for treating human infections, but also in livestock and fish farms. As a consequence, large quantities of these antimicrobials are released into aquatic environments via sewage or directly from livestock and fish farms, thereby providing a selective pressure for the development or acquisition of antimicrobial-resistant bacteria (ARB) and genes (ARGs) (Esteve et al., 2015). Indeed, aquatic environments have been shown to function as reservoirs of ARB and ARGs (Marti et al., 2014), and the transfer of plasmid-encoded ARGs from aquatic environments to humans comprises the greatest potential risk to human health (Smith et al., 1994). To date, however, there have been few reports regarding the monitoring of antimicrobial resistance in aquatic environments.

To efficiently monitor antimicrobial resistance, it is necessary to identify an effective general indicator bacterium within aquatic environments. For example, *Escherichia coli* is commonly used to monitor antimicrobial resistance in clinical settings and in the veterinary field (NARMS, 2012; National Veterinary Assay Laboratory Forestry and Fisheries, 2012); however, this organism is not always isolated from aquatic environments. In contrast, *Aeromonas* spp. are Gram-negative, waterborne organisms that are ubiquitous in most aquatic environments (Rhodes et al., 2000). We therefore selected *Aeromonas* spp. as a general model organism to inform on antimicrobial resistance in aquatic environments.

The spread and maintenance of ARB and ARGs in aquatic environments comprises a potential human health risk. In Southeastern Asian countries, including Thailand, human habitats are often located in close proximity to aquatic environments. Indeed, contamination of both humans and livestock animals with water-derived ARB has been demonstrated in tropical Asian countries (Shimizu et al., 2013). Likewise, a previous study detected contamination of individuals in Indochina with ARGs derived from aquatic environments (Suzuki and Hoa, 2012). Thailand is one of the most agriculturally productive countries in Southeast Asia, and livestock animals in this country are frequently administered antimicrobials to treat and prevent infections (Daniel et al., 2015). As a result, individuals in Thailand are likely at a greater risk of exposure to ARB and ARGs than those in other countries in this region. In this study, we first demonstrated the usefulness of *Aeromonas* spp. as general indicator bacteria for monitoring antimicrobial resistance. We subsequently utilized this organism to evaluate the current prevalence of resistance at multiple aquatic sites in Thailand. Finally, we examined putative correlations between the prevalence of ARB and ARGs and drug concentrations in rivers and livestock wastewater.

## Materials and Methods

### Sample Collection

**FIGURE 1:**
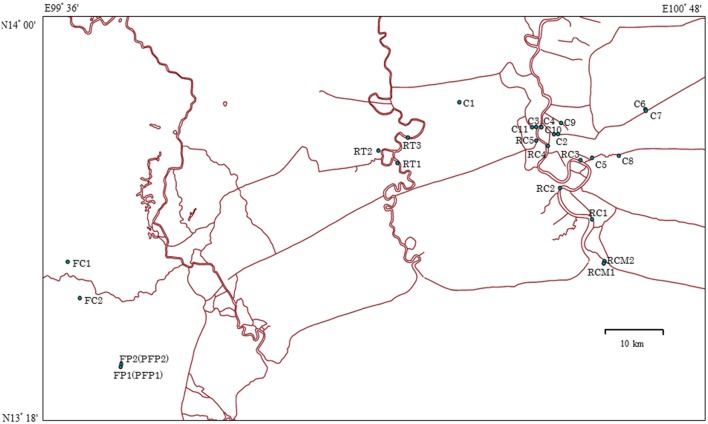
**Map of sampling sites in Thailand.** RC 1–5 indicate Chao Phraya River sites; RCM 1 and 2 indicate sites at the mouth of Chao Phraya River; RT 1–3 indicate Ta Chin River sites; C 1–5 indicate the city canal sites sampled in 2013; C 6–11 indicate the city canal sites sampled in 2014; FC 1 and 2 and FP 1 and 2 indicate chicken and pig farm sites, respectively. Sources: http://www.savgis.org/cv_EN.htm.

Grab water samples were collected in sterile 1-L bottles in Bangkok and in the province of Ratchaburi in September 2013 and 2014 (**Figure 1**). The characteristics of the sampling sites are summarized in **Supplementary Table S1**. Specifically, in 2013, surface water samples were taken at 5 sites (RC 1, 2, 3, 4, and 5) on the Chao Phraya River and at 2 sites (RCM 1 and 2) at the mouth of Chao Phraya River. In addition, 3 sites (RT 1, 2, and 3) were sampled on the Ta Chin River and 5 samples were harvested at city canals (C 1, 2, 3, 4, and 5) in Bangkok. In 2014, 6 samples were taken at city canals (C 6, 7, 8, 9, 10, and 11) in Bangkok, and effluent was collected from chicken farms (FC 1 and 2) and pig farms (FP 1 and 2) in the province of Ratchaburi. The pig farm effluent was also treated at a biogas plant. Treated effluent is indicated as post-treatment FP 1 and 2 (FFP1 and FFP2). All samples were stored at 4°C after collection and analyzed within 12 h.

### Analysis of Antimicrobial Concentrations

To measure aquatic concentrations of antimicrobials, including sulfonamides and tetracyclines, water samples were analyzed using a liquid chromatograph (Accela, Thermo Scientific, Waltham, MA, USA) equipped with a tandem mass spectrometer (LC-MS/MS; Quantum Access, Thermo Scientific) after extraction using a solid-phase cartridge (Oasis HLB resin; Waters Corp., Milford, MA, USA). The analytical process was the same as that described by Segura et al. (2015).

### Bacterial Isolation

For isolation of *Aeromonas* spp. from water samples, deoxycholate-hydrogen sulfate-lactose (DHL) agar medium (Nissui Pharmaceutical, Tokyo, Japan) was inoculated with 100 μL of each respective sample and incubated at 37°C overnight. For each sample, a maximum of six colonies were identified as likely *Aeromonas* spp. based on colony morphology on DHL agar (pink colony) and were subjected to further analyses. Specifically, the isolates were screened for oxidase production and subjected to a variety of biochemical tests (API20E; BioMérieux, Marcy l’Etoile, France). Furthermore, the identity of each strain was verified by 16S rRNA gene sequencing (Marchesi et al., 1998).

### Antimicrobial Susceptibility Testing

The minimal inhibitory concentrations (MICs) of the following antimicrobials for each isolate were determined using the agar dilution method, according to the Clinical Laboratory Standards Institute (CLSI) guidelines (Clinical Laboratory Standards Institute, 2008): ampicillin, cefazolin, cefotaxime, kanamycin, tetracycline, sulfamethoxazole, nalidixic acid, and ciprofloxacin (all obtained from Sigma-Aldrich, St. Louis, MO, USA). The breakpoints for each antimicrobial were in accordance with CLSI guidelines (Clinical Laboratory Standards Institute, 2008), except for those of sulfamethoxazole, which are not defined by the CLSI guidelines. As such, we utilized the break points for sulfonamide for this antimicrobial. *E. coli* ATCC25922, *Staphylococcus aureus* ATCC29213, *Enterococcus faecalis* ATCC29212, and *Pseudomonas aeruginosa* ATCC27853 were used as quality control strains.

### Characterization of ARGs

Each strain was evaluated for the presence of the *tetA*, *tetB*, *tetC*, *tetD*, *tetE*, and *tetG* genes via multiplex PCR, as previously described (Jin Jun et al., 2004). Meanwhile, PCR analysis was utilized to detect the *tetM* and *tetS* genes (Kim et al., 2004) and the *sul1*, *sul2*, and *sul3* genes (Hoa et al., 2008), respectively.

### Statistical Analysis

Correlations between the environmental concentration of each antimicrobial and both the rate of antimicrobial resistance and the prevalence of resistance genes among *Aeromonas* spp., respectively, were evaluated using Pearson’s test. Differences were considered significant at *p* < 0.05.

## Results

### Isolation of *Aeromonas* spp. from Water Samples

In total, 117 *Aeromonas* strains were isolated from 27 water samples. Specifically, 24, 9, 13, 13, 30, 9, 12, and 7 strains were isolated from riverine of Chao Phraya River, the mouth of Chao Phraya River, the riverine of Ta Chin River, the city canal, chicken farms, pig farms, and post-treatment pig farms, respectively (**Table 1**).

**Table 1:** Prevalence of antimicrobial resistance genes (ARGs) among *Aeromonas* spp. isolated from aquatic environments in Thailand.

		Tetracycline resistance genes						Sulfonamide resistance genes		
				
Sampling sites	*n*	*tetA* (%)	*tetB* (%)	*tetC* (%)	*tetE* (%)	*tetM* (%)		*sul1* (%)	*sul2* (%)	*sul3* (%)
Chao Phraya River	24	4.2	0	8.3	29.1	0		37.5	0	0
Mouth of the Chao Phraya River	9	11.1	0	11.1	33.3	0		44.4	0	0
Ta Chin River	13	0	0	15.4	0	0		15.4	0	0
City Canal, 2013	13	7.7	0	7.7	7.7	0		30.8	0	0
City Canal, 2014	30	0	3.3	20	23.3	0		26.7	3.3	0
Chicken Farm	9	0	0	0	0	0		0	0	0
Pig Farm	12	75	0	0	0	16.7		25	33.3	25
Pig Farm, Post-treatment	7	28.5	0	0	0	14.3		28.6	42.9	14.3


### Antimicrobial Resistance

**FIGURE 2:**
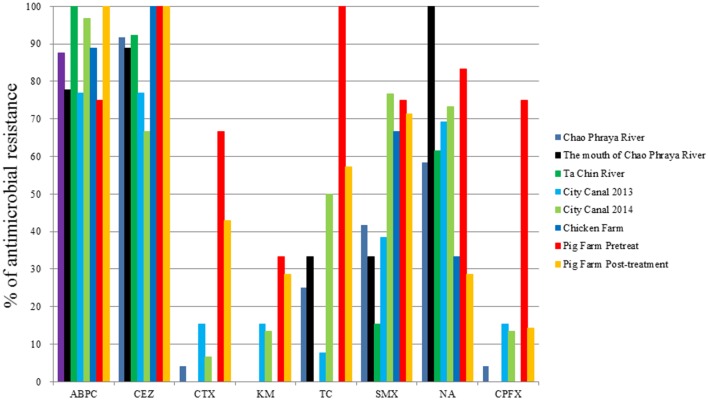
**Antimicrobial resistance rates of *Aeromonas* spp. obtained from several aquatic environments.** ABPC, ampicillin; CEZ, cefazolin; CTX, cefotaxime; KM, kanamycin; TC, tetracycline; SMX, sulfamethoxazole; NA, nalidixic acid; CPFX, ciprofloxacin.

The proportions of antimicrobial-resistant *Aeromonas* spp. are summarized in **Figure 2.** Nearly all isolates were resistant to ampicillin and cefazolin. Likewise, we frequently detected sulfamethoxazole- and nalidixic acid-resistant isolates. Notably, however, the rates of cefotaxime, kanamycin, tetracycline, and ciprofloxacin resistance were markedly higher among isolates derived from pig farms than among those obtained from other sites.

### Antimicrobial Resistance Genes (ARGs)

While the *tetA* gene was detected in multiple river and canal samples, the prevalence of this gene was highest among the pig farm isolates (75.0%; **Table 1**). Moreover, both the *tetM* and *sul3* genes (16.7 and 25%, respectively; **Table 1**) were detected only in isolates harvested from pig farms, while the *sul2* gene was detected in pig farm and canal (2014) isolates only (33.3 and 3.3%, respectively; **Table 1**). The *tetC* and *tetE* genes were the most frequently detected resistance genes among the river isolates (**Table 1**), and the *sul1* gene was detected in all water environments except for chicken farms (15.4 to 44.4%). Notably, the *tetC, tetG*, and *tetS* genes were not detected in any of the isolates examined in this study, while *tetB* was detected in only 1 of the 30 canal isolates from 2014 (3.3%).

### Relationship between Rates of Antimicrobial Resistance and Concentrations of Antimicrobials

**FIGURE 3:**
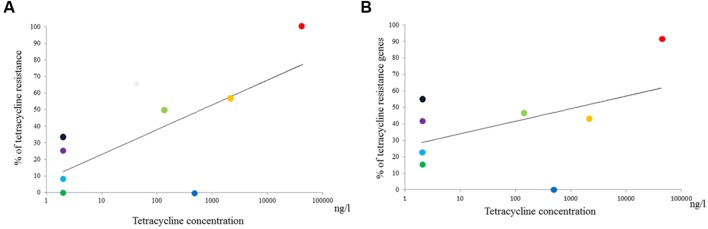
**Relationship between the tetracycline concentration at individual aquatic environments and **(A)** rates of tetracycline resistance or **(B)** the prevalence of tetracycline resistance genes among *Aeromonas* spp, respectively.** Purple, Chao Phraya River; Black, The mouth of Chao Phraya River; Green, Ta Chin River; Sky Blue, City canal 2013; Yellow-Green, City canal 2014; Blue, Chicken farm; Red, Pig farm; Orange, Pig farm post-treatment.

The average concentrations of tetracyclines and sulfonamides at each sample site are summarized in **Table 2.** Subsequent statistical analyses detected significant correlations between the environmental concentration of tetracyclines and both the rate of tetracycline resistance and the prevalence of tetracycline resistance genes among *Aeromonas* spp. (*R*^2^ = 0.63 and *R*^2^ = 0.57, *p* = 0.019 and *p* = 0.030, respectively; **Figure 3**). In contrast, there was no statistical correlation between the environmental concentration of sulfonamides and the rate of sulfamethoxazole resistance or the prevalence of sulfonamide resistance genes among *Aeromonas* spp.

**Table 2:** The average concentrations of tetracyclines and sulfonamides at the aquatic environments examined in this study.

		Average concentrations (ng/L)	
		
Sampling sites	*n*	Tetracyclines	Sulfonamides
Chao Phraya River	5	<2	12.6 ± 5.0
Mouth of the Chao Phraya River	2	<2	13.7 ± 2.4
Ta Chin River	3	<2	33.7 ± 1.9
City Canal, 2013	5	<2	74.8 ± 54.8
City Canal, 2014	6	138.8 ± 123.2	111.5 ± 119.2
Chicken Farm	2	482.0 ± 503.5	ND
Pig Farm	2	43,500.0 ± 4,0527.1	48.0 ± 9.9
Pig Farm, Post-treatment	2	2,135.5 ± 365.6	12.5 ± 16.3


*ND, not detected.*

## Discussion

In this study, *Aeromonas* spp. were frequently isolated from water samples. Notably, these isolates exhibited various resistance patterns against the antimicrobials tested, with the exception of ampicillin and cefazolin, to which nearly all strains were resistant. These findings are therefore consistent with those of a previous study demonstrating that *Aeromonas* spp. are endogenously resistant to these two antimicrobials (Cisar et al., 2014). Meanwhile, the cephalosporin-resistant *Aeromonas* spp. exhibited resistance against one of the latest generation of cephalosporins, cefotaxime. As such, monitoring of cefotaxime resistance in *Aeromonas* spp. would provide information regarding the resistance rates to latest generation cephalosporins among aquatic isolates. Furthermore, Yano et al. (2015) previously reported that *Aeromonas* spp. exhibited a greater variety of antimicrobial susceptibility profiles than other aquatic bacteria (Yano et al., 2015). Together, these results suggest that *Aeromonas* spp. comprise an effective general indicator organism for monitoring antimicrobial resistance in aquatic environments.

*Aeromonas* spp. were previously found to be as prevalent in humans as in environmental sources, indicating that these organisms could disseminate antimicrobial resistance from environmental to clinical environments (Sanchez-Cespedes et al., 2009). Meanwhile, yet-to-be cultured bacteria in aquatic environments can act as non-visible ARG reservoirs (Suzuki et al., 2015), and may be capable of transferring these ARGs to both *Aeromonas* spp. and other pathogenic aquatic bacteria. As such, monitoring the prevalence and dissemination of antimicrobial resistance in aquatic environments using *Aeromonas* spp. as an indicator organism is essential to minimize potential risks to human health.

In this study, we detected 40-fold higher concentrations of tetracyclines in pig farm wastewater than in river water samples. Indeed, in the two pig farms tested, chlortetracycline was used as a feed additive for 15- to 20-week-old piglets (personal communications). In contrast, the concentrations of sulfonamides detected in this study were markedly lower than those previously detected on pig farms in Vietnam and South Africa (Le and Munekage, 2004; Hoa et al., 2011; Shimizu et al., 2013; Suzuki et al., 2015). These results suggest that while tetracyclines are currently used intensively, sulfonamides have begun to be phased out for agricultural use in Thailand.

We detected a significant correlation between the environmental concentration of tetracyclines and the rate of tetracycline resistance, but not between the concentration of sulfonamides and the rate of sulfonamide resistance among the *Aeromonas* strains isolated in this study. Our findings are therefore partially consistent with those of a previous study, which reported that high concentrations of antimicrobials (ciprofloxacin, ofloxacin, cefazolin, cefotaxime, sulfamethoxazole, and clarithromycin) were associated with high copy numbers of ARGs in hospital wastewater samples (Rodriguez-Mozaz et al., 2015). Indeed, these high concentrations of antimicrobials played an important role in the selection and maintenance of ARB and ARGs. These findings therefore suggest that aquatic environments, particularly those containing high concentrations of antimicrobials, act as reservoirs of ARB and ARGs. However, our sampling data were limited, especially in regard to sampling points associated with high concentrations of antimicrobials. As such, in future studies, a greater number of such sampling points is needed to obtain more reliable findings.

The *tetA* gene was the most frequently detected ARG among tetracycline-resistant *Aeromonas* isolates derived from pig farms. In a previous report, *tetA* was the most prevalent tetracycline-resistant gene among *E. coli* strains isolated from pig feces (Schwaiger et al., 2010), suggesting that the *tetA* gene of *Aeromonas* spp. isolated from pig farm wastewater originated from pig feces. Meanwhile, the *tetE* gene was the mostly frequently observed ARG among isolates derived from river and canal water. Notably, *tetE* has also been detected in aquatic sediments from aqua culture farms, despite the absence of tetracycline treatments (Tamminen et al., 2011). In most aquatic environments, antimicrobial concentrations are low [e.g., sub- to low-μg/L levels, even in anthropogenically impacted waters (Shimizu et al., 2013)]. However, long-term consistent exposure to sub-threshold doses of antimicrobials can result in increased rates of bacterial resistance (Harnisz, 2013). These results suggest that antimicrobial resistance genes (ARGs), including *tetE*, can be maintained in aquatic environments regardless of the concentration of the corresponding antimicrobial. Consistent with this conclusion, we recently demonstrated that multidrug resistance plasmids can be retained without selective pressure in viable but non-culturable cells in oligotrophic water environments (Bien et al., 2015).

In this study, treatment of pig farm samples at a biogas plant resulted in decreased rates of resistance to six antimicrobials, as well as reduced concentrations of antimicrobials. Likewise, a previous study demonstrated that biogas plant treatments decrease the prevalence of ARB and tetracyclines in cattle feces (Ihara et al., 2012). These results suggest that biogas plant treatments comprise an effective method for decreasing the concentration of certain antimicrobials and ARB in livestock wastewater.

## Author Contributions

Study conception and design: MU, CB, SS, HT, YT. Acquisition of data: MU, CT, AF, TO, KS, HT. Analysis and interpretation of data: MU, CT, SS, HT, YT. Drafting of manuscript: MU, SS, HT, YT.

## Conflict of Interest Statement

The authors declare that the research was conducted in the absence of any commercial or financial relationships that could be construed as a potential conflict of interest.
