# Supplementary material for: Use of Aeromonas spp. as General Indicators of Antimicrobial Susceptibility among Bacteria in Aquatic Environments in Thailand
Source: Front Microbiol. 2016 May 12;7:710. doi: 10.3389/fmicb.2016.00710 (PMC4922218; doi:10.3389/fmicb.2016.00710)
Supplement: Supplementary file 1 [file Table_1.DOCX]

Supplementary Material

Use of *Aeromonas* spp. as general indicators of antimicrobial susceptibility among bacteria in aquatic environments in Thailand

Masaru Usui^1^, Chie Tagaki^1^, Akira Fukuda^1^, Torahiko Okubo^1^, Chanchai Boonla^2^, Satoru Suzuki^3^, Hideshige Takada^4^, and Yutaka Tamura^1*^

^1^School of Veterinary Medicine, Rakuno Gakuen University, Ebetsu, Hokkaido, Japan

^2^Departments of Biochemistry, Chulalongkorn University, Bangkok, Thailand

^3^Center for Marine Environmental Studies, Ehime University, Matsuyama, Ehime, Japan

^4^Laboratory of Organic Geochemistry, Tokyo University of Agriculture and Technology, Fuchu, Tokyo, Japan

*Correspondence: Yutaka Tamura, D.V.M., Ph.D.

## E-mail address: [tamuray@rakuno.ac.jp](mailto:tamuray@rakuno.ac.jp)

## Supplementary Tables

**Supplementary Table 1. Characteristics of the sites sampled in this study**

| Site | River name | Water temp (°C) | pH | EC (µS/cm) | Salinity | Depth (m) | Description |
| --- | --- | --- | --- | --- | --- | --- | --- |
| RC 1 | Chao Phraya River | 28.7 | 7.44 | 28.5 | 0 | 10 |  |
| RC 2 | Chao Phraya River | 28.9 | 7.38 | 25.1 | 0 | 3 |  |
| RC 3 | Chao Phraya River | 28.9 | 7.31 | 24.8 | 0 | nd |  |
| RC 4 | Chao Phraya River | 28.7 | 7.52 | 25.8 | 0 | 3 |  |
| RC 5 | Chao Phraya River | 28.8 | 7.48 | 23.7 | 0 | 5 |  |
| RCM 1 | Chao Phraya River | 28.7 | 7.42 | 157 | 0 | 6 |  |
| RCM 2 | Chao Phraya River | 28.6 | 7.39 | 97.1 | 0.5 | 6 |  |
| RT 1 | Ta Chin River | **28.8** | 7.33 | 45.7 | 0 | nd |  |
| RT 2 | Ta Chin River | 28.6 | 7.32 | 44.6 | 0 | 4 |  |
| RT 3 | Ta Chin River | 28.2 | 7.3 | 41.4 | 0 | nd |  |
| C 1 | Unknown | 29.4 | 7.38 | 72.1 | 0 | 1.5 | A canal in Bangkok. |
| C 2 | Unknown | 29.1 | 7.41 | 35.1 | 0 | nd | A canal in Bangkok. |
| C 3 | Unknown | 29.4 | 7.62 | 25.4 | 0 | 4 | A canal in Bangkok. |
| C 4 | Unknown | 29.1 | 7.62 | 22.4 | 0 | 4 | A canal in Bangkok. |
| C 5 | Unknown | 28.7 | 7.3 | 66.9 | 0 | 2 | A canal in Bangkok. |
| C 6 | Unknown | 31.7 | 7.29 | 646 | nd | d | A canal in Bangkok. |
| C 7 | Unknown | 31.5 | 7.28 | 593 | nd | nd | A river near TBC1. |
| C 8 | Unknown | 33.1 | 7.41 | 798 | nd | nd | A narrow canal beside the street. |
| C 9 | Unknown | 32.1 | 7.30 | 615 | nd | nd | A narrow canal behind the temple. |
| C 10 | Unknown | 31.9 | 7.37 | 503 | nd | nd | A narrow canal. |
| C 11 | Unknown | 32.7 | 7.55 | 500 | nd | nd | A narrow canal. |
| FC 1 | Unknown | 30.9 | 8.70 | 296 | nd | nd | Wastewater from a chicken house. |
| FC 2 | Unknown | 29.5 | 7.37 | 366 | nd | nd | Wastewater from a chicken house. |
| FP 1 | Unknown | 30.7 | 8.14 | 6,030 | nd | nd | Wastewater harvested from a pig house prior to treatment. |
| FP 2 | Unknown | 30.5 | 8.10 | 4,310 | nd | nd | Wastewater harvested from a pig house prior to treatment. |
| PFP 1 | Unknown | 29.1 | 8.02 | 4,040 | nd | nd | Wastewater harvested from a pig house post-treatment |
| PFP 2 | Unknown | 32.3 | 8.20 | 7,010 | nd | nd | Wastewater harvested from a pig house post-treatment |

nd, not determined
